# Extravascular FIX and coagulation

**DOI:** 10.1186/s12959-016-0104-2

**Published:** 2016-10-04

**Authors:** Darrel W. Stafford

**Affiliations:** Biology and Pathology, University of North Carolina at Chapel Hill, Chapel Hill, NC 27599-3280 USA

**Keywords:** FIX, Collagen IV, Coagulation, Hemophilia B

## Abstract

This review summarizes the evidence that collagen IV binding is physiologically important, and that the extravascular compartment of FIX is composed of type IV collagen. As we have previously demonstrated, 7 days post-infusion, FIX_WT_ (BeneFIX) is able to control bleeding as well as the same dosage of Alprolix in hemophilia B mice, tested using the saphenous vein bleeding model (Alprolix is a chimeric FIX molecule joined at its C terminus to a Fc domain). Furthermore, we have shown that in hemophilia B mice, doses of BeneFIX or Alprolix (up to a dose of 150 IU/kg) have increased bleeding-control effectiveness in proportion to the dose up to a certain limit: higher doses are no more effective than the 150 IU/kg dose. These studies suggest that in hemophilia B mice, tested using the saphenous vein bleeding model, three things are true: first, extravascular FIX is at least as important for coagulation as is circulating FIX; second, measuring circulating levels of FIX may not be the best criterion for designing new “longer lasting” FIX molecules; and third, trough levels are less diagnostic for FIX therapy than they are for FVIII therapy.

## Background

Hemophilia B is caused by the absence or defect of an enzyme, coagulation factor IX (FIX). A plasma concentration of ~5 μg per ml of FIX, defined to be one international unit (IU), is the approximate average plasma concentration of pooled samples from normal individuals.

Familial bleeding, or hemophilia, has been known since biblical times. In 1952, several groups realized that the blood of some hemophilia patients could correct coagulation in blood from other hemophilia patients [1–3]. This observation led to the designation of two types of hemophilia: hemophilia A (FVIII deficiency) and hemophilia B (FIX deficiency), which are clinically indistinguishable. The designation of disease severity has been well defined for hemophilia A. Generally, patients who have less than 1 % of the normal circulating levels of FVIII in their plasma are designated as severe cases and have frequent occurrences of spontaneous bleeding. Patients with between 1 and 5 % activity are designated moderate cases; these patients experience bleeding primarily after injury. Patients with levels between 5 and 50 % activity are considered to have mild hemophilia and usually experience bleeds only with surgery or major trauma. Although the same designation criteria are applied to hemophilia B, the correlation between plasma levels and clinical outcome has not been rigorously proven [4].

Before the advent of replacement therapy, the average life span for a severe hemophilia patient was 13 years. A monograph published in 1937 [5] examined the fate of 98 severe hemophilia patients; 83 died before age 16, and only 6 survived to age 40. Further, 24 of the 98 died from minor surgery or insignificant injuries, such as biting their tongue or cutting a finger. After the development of specific purified or recombinant FIX and FVIII molecules for replacement therapy, the average age of survival of hemophilia patients increased to about 60 years.

The standard of care has, until recently, been to treat patients for severe bleeding episodes as they occur (on demand). Recently, however, the World Federation of Hemophilia recommends that on-demand treatment be replaced by prophylaxis [6]. The goal is to prevent osteoarthritis caused by bleeding into joints.

Currently, clinical practice guidelines for prophylactic hemophilia treatment requires 2–3 infusions per week. However, several companies have developed new, “longer lasting” FIX molecules; these have been designed to have a longer plasma half-life, and thus theoretically allow patients to go 7–10 days between injections [7–9].

**Fig. 1:**
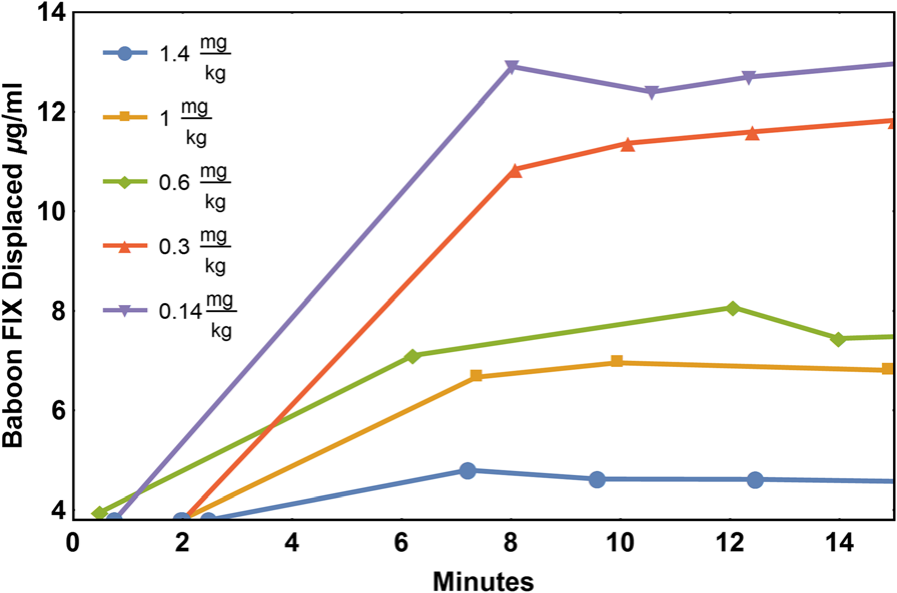
Comparison of the ability of different concentrations of bovine factor IX to displace baboon factor IX from its extravascular pool. Redrawn from Stern et al. Br J. Haemat. 66:227. (1987). Adult baboons were infused with the indicated amounts of bovine FIX, and the amount of baboon FIX in the plasma was measured over the first 15 min post-infusion

While the published half-life data on these “longer lasting” molecules is impressive, the extravascular role of FIX has not been considered. It is standard clinical practice to treat hemophilia as though the plasma concentration of FIX is the only significant criterion. However, this ignores the significant evidence for the existence of extravascular FIX and the role that it plays in coagulation. The following observations provide evidence that the naturally occurring extravascular stores of FIX are physiologically important. First, FIX binds reversibly and saturably to type IV collagen [10]. Second, a mouse expressing human FIX (FIX_K5A_, which has a specific activity in vitro of up to twofold greater than that of FIX_WT_, but a reduced affinity for type IV collagen) has a bleeding diathesis, even though its circulating level of FIX_K5A_ is about 20 % higher than normal [11]; the mouse exhibits this bleeding diathesis although both the plasma levels and specific activity are higher for the FIX in the FIX_K5A_ mouse. Third, we infused hemophilia B mice with a FIX variant (FIX_K5R_) that binds more tightly to type IV collagen than does FIX_WT._ The mice dosed with FIX_K5R_ clotted more efficiently mice infused with FIX_WT_; this occurred despite the fact that circulating FIX levels were unmeasurable 3 days post-infusion [12] . There is also excellent evidence of an extravascular reservoir of FIX in baboons. Stern et al. demonstrated quite clearly that administering increasing amounts of bovine FIX into the baboon rapidly displaced endogenous stores of extravascular baboon FIX into the plasma [13]. In Fig. 1, Stern et al. infused baboons with bovine FIX and then followed the release of baboon FIX into the plasma. This was possible due to a specific antibody for bovine FIX that allows the measurement of baboon FIX with minimal cross-reactivity with bovine FIX. Clearly, in the baboon experiments, at least 3 times more FIX is released, presumably from an extravascular collagen IV compartment, than is found in the baboons’ plasma.

**Fig. 2:**
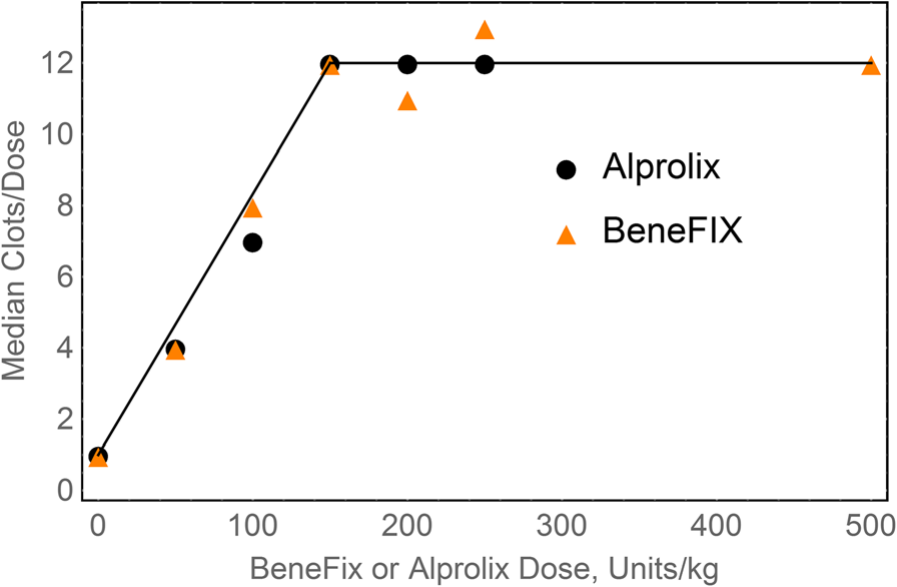
Comparison of different doses of factor IX to effect hemostasis seven days post-infusion in hemophilia B mice. From: Cooley et al. Blood. 128:286. (2016). Doses of 50, 100, 150, 250, and 500 IU/kg of either Alprolix or Benefix were infused into hemophilia B mice. After 7 days, the ability of the mice to maintain hemostasis was evaluated by the saphenous vein bleeding model. At least 20 mice were used for each dose

Figure 2 shows that, at doses of 50, 100 and 150 IU/kg, Alprolix and BeneFIX respond almost identically in hemophilia B mice tested using a saphenous vein bleeding model, and that maximum efficacy is achieved at a dose of about 150 IU/kg. Figure 2 shows that, for optimal long-term therapy, 150 IU/kg is best, at least in mice. In Fig. 2, hemophilia B mice were infused with BeneFIX at doses of 50, 100, 150, 200, 250, and 500 IU/kg and Alprolix at doses of 50, 100, 150 and 250 IU/kg. These data were tested using a saphenous vein bleeding model and were, for each dose and molecule, observed 7 days post-infusion.

**Fig. 3:**
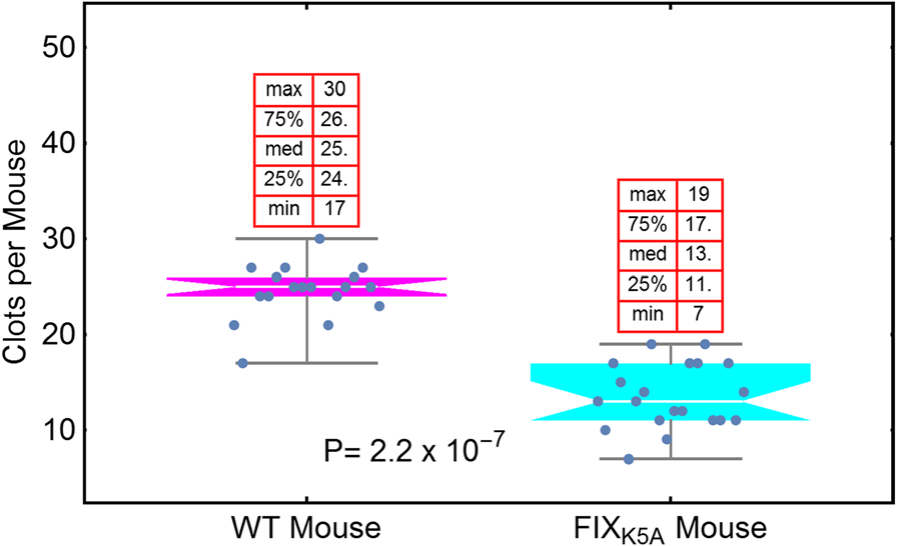
Comparison of the hemostatic ability of wild-type mice and hemophilia B mice whose endogenous factor IX was exchanged for FIX_K5A_. From: Cooley et al. Blood. 128:286. (2016). A mouse expressing FIX_K5A_ from its endogenous promoter was compared to a wild-type mouse using the saphenous vein bleeding model. The activity level of the FIX_K5A_ in the plasma of this mouse was slightly higher than that of the wild-type mouse. The mouse expressing FIX_K5A_ nevertheless has a bleeding diathesis

We utilized homologous recombination to replace defective mouse FIX in our hemophilia B mice [14], creating the FIX_K5A_ mouse, which has a reduced FIX affinity for collagen IV [15]. This FIX_K5A_ mouse has a mild bleeding diathesis, although it has 120 % of the circulating FIX_K5A_ of WT mice—even though the FIX_K5R_ displays about a twofold increase in specific activity over the FIX_WT_ mouse in an APTT assay [16, 17]. Figure 3 shows the efficacy of FIX_WT_ and FIX_K5A_ in mice (data taken using the saphenous vein model). Clearly there is a bleeding diathesis associated with reduced FIX affinity for collagen IV, even though the FIX plasma activity in these mice is higher than in the wild-type mice.

**Fig. 4:**
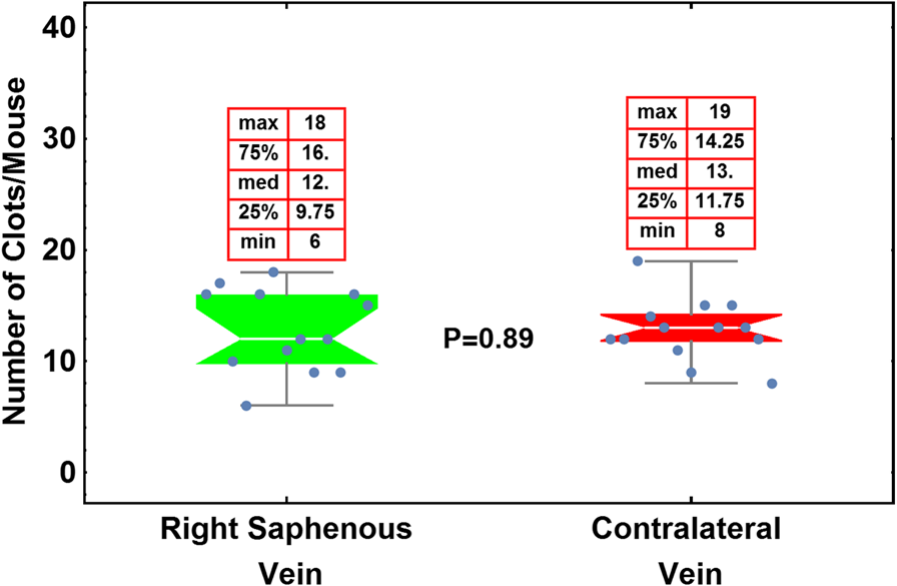
An examination of the ability of FIX (BeneFIX) to effect hemostasis 7 days post-infusion, after the ability of a first injury to effect clotting has been exhausted. From: Cooley et al. Blood. 128:286. (2016). Seven days post-infusion, the right saphenous vein was injured and the number of times that re-clotting ensued was determined. After the first injury was unable to continue clotting, the contralateral vein was injured and hemostatic efficacy was again measured. Presumably, any residual plasma FIX would have been used up during the first injury had plasma FIX been required for clotting

What causes this cessation of bleeding when FIX is at immeasurable levels in the plasma? Is it residual FIX circulating FIX that is below the limits of detection but still effective? Is it collagen IV-bound, extravascular FIX? We examined this question recently in a paper published in *Blood* [18]. First we performed a saphenous vein injury on one side of the mouse and measured how many times bleeding could be terminated. Any unmeasurable, residual circulating FIX should have been exhausted when the first vein lost the ability to clot. We then injured the contralateral vein and measured the number of times that clotting from the injury ceases the bleeding. The clotting efficacy for the contralateral vein was essentially identical to that of the initial injury (Fig. 4). This appears to mean that the local extravascular FIX, not the circulating FIX, is critical for the cessation of bleeding—at least in this model of coagulation efficacy.

### Are FIX’s pharmacokinetics consistent with rates of disappearance from plasma?

**Fig. 5:**
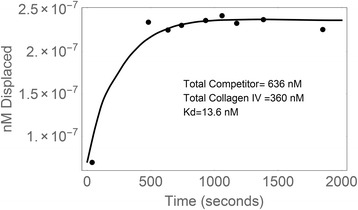
A fit of Stern’s data [Stern et al. Br J. Haemat. 66:227. (1987)] to a simple set of differential equations describing equilibrium. Stern’s data was digitized and fit to a simple differential equation with the assumption that, during the first 10 min after infusion, only equilibrium between FIX and type IV collagen occurs. The numbers are at least consistent with in vitro measurements of the binding constants between FIX and collagen IV

One question that is frequently asked is: what about the on and off rates for proteins? Are they really consistent with FIX binding to collagen IV? It is very difficult to obtain this data, and the limited number of data points make the results approximate. But when Stern’s data is fitted to a simple differential equation that assumes that the only thing going on in the first 10 min post-infusion is binding to collagen IV, the resulting numbers are consistent with the in-vitro measured K_D_ values of around 5 nM (Fig. 5). For example, in Stern’s data, the rate of displacement of baboon FIX into the plasma is around 0.003 per second, while the association rate constant is about 3 × 10^5^ per second per mole. These values are typical on and off rates for proteins [19].

### How much extravascular FIX is expected?

If we assume that FIX’s binding affinity for collagen IV is 5 nM and that the FIX plasma concentration is 90 nM, then approximately 95 % of the total FIX will be bound to available type IV collagen. This predicts that about 20-fold more extravascular FIX is present outside the vasculature than is found in the circulation. This would be true unless the collagen IV were limiting. While it is difficult to estimate the total amount of collagen IV in the body, it is likely that there is much more collagen IV than would be saturated by our proposed optimal dosage of 150 IU/kg of FIX (if all of the infused 150 IU/kg FIX were retained in the plasma, the molar concentration of FIX would be about 340 nM). Saturation of the extravascular sites at concentrations of FIX of around 300 to 400 nM agrees very well with the values necessary to get kinetic constants with pharmacokinetic data. The 300 to 400 nM number also agrees with Stern’s data on the displacement of baboon FIX by bovine FIX with theoretical plots.

### How can we explain the lack of binding of FIX to much of the available collagen IV?

Much of the body’s collagen IV is probably not normally in contact with plasma-borne FIX. But the sinusoids, which do not bind FIX, clearly are exposed to the circulation. A possible explanation for this discrepancy is that post-translational modification of collagen IV is required to prevent binding of FIX to regions of collagen IV that are readily accessible to blood. An example is provided recently by a report that a specific prolyl hydroxylase is required to prevent lethal platelet aggregation via the platelet receptor GPVI [20]. A similar post-translational modification could render the collagen IV lining the sinusoids incapable of binding with FIX.

### Thrombosis at higher doses

The best evidence against a slight elevation of FIX causing a significant risk of thrombosis comes from a particular group of patients whose FIX—FIX Padua (FIX_R333L_)—has a specific activity that is about eight times greater than that of FIX_WT_ [21]. Affected males develop thromboses at around 16–20 years of age, but the heterozygous mother, whose FIX activity is increased to almost 4 times that of FIX_WT_, has had no problems with thrombosis. The FIX Padua patients in the Simioni study are chronically exposed to FIX that, while present at normal doses, has an eightfold increase in specific activity, which should be equivalent to the activity of 8 times the normal amount of circulating FIX. By contrast, hemophilia B patients infused with 150 IU/kg of FIX would be only transiently exposed to higher doses of circulating FIX. For example, in our experiments that injected different amounts of FIX into hemophilia B mice, we found that at a dose of 150 IU/kg, the increased level of plasma exposure lasts for less than 2 min. Even at doses of 320 IU/kg, the plasma level of FIX decreases to normal levels in mice by the 30-min mark. More importantly, the studies that correlate circulating FIX activity to thrombosis risk do not consider the relatively large extravascular reservoir of FIX. For example, a plasma level of 5 mcg/mL would imply a total dose of FIX of at least 22.5 mcg/mL—equivalent to a dose of about 180 IU/kg of FIX. So a dose of 150–200 IU/kg given to a patient with no circulating levels of FIX antigen is very nearly the same (or less) than the total amount present in normal individuals. Clearly, not all the loss of FIX from circulation is caused by clearance in the traditional sense, because infused hemophilia B mice continue to exhibit relatively good function—even 7 days post-infusion, when no FIX has been detectible in the circulation since day 3 post-infusion. In mice, Alprolix and BeneFIX protect from bleeding for 7 days with essentially identical efficacy.

## Conclusions

The cumulative thrust of these studies sheds light on the reports that, at the same plasma levels of activity, FVIII mutations appear to be more severe than FIX mutations expressing the same levels of FIX activity [22, 23]. This difference may be explained by the role of extravascular FIX in coagulation—a significant proportion of FIX is extravascular, and this extravascular FIX remains active. Presumably, FVIII does not have significant extravascular stores.

The assumption that plasma FIX concentrations are the best marker of FIX supplementation efficacy is thus not completely true. FIX is also found distributed around the extra-vasculature throughout the body. The sites that react with antibodies to FIX also react with antibodies to type IV collagen (although not all of the apparently readily available collagen IV in the body reacts with FIX antibodies).

Accumulating data suggest that the extravascular compartment approaches saturation at around 150 IU/kg; therefore we suggest that, an appropriate dose of FIX would be around 150 IU/kg. Longer time points should also be tested in future experiments, since, at this dose, there is still rather robust control at 7 days post-infusion.

## Abbreviations

FIX: Coagulation FIX
Hemophilia B: an individual or animal lacking FIX
